# Biodiversity and Geographic Distribution of Rhizobia Nodulating With *Vigna minima*

**DOI:** 10.3389/fmicb.2021.665839

**Published:** 2021-05-04

**Authors:** Guohua Liu, Xiaoling Liu, Wei Liu, Kangning Gao, Xiaoli Chen, En-Tao Wang, Zhenjun Zhao, Wenxiao Du, Yan Li

**Affiliations:** ^1^College of Life Science, Yantai University, Yantai, China; ^2^Key Laboratory of Coastal Biology and Bioresource Utilization, Yantai Institute of Coastal Zone Research, Chinese Academy of Sciences, Yantai, China; ^3^College of Resources and Environment, Shijiazhuang University, Shijiazhuang, China; ^4^The Fruit Trees Work Station of Penglai, Yantai, China; ^5^Departamento de Microbiología, Escuela Nacional de Ciencias Biológicas, Instituto Politécnico Nacional, Mexico City, Mexico

**Keywords:** rhizobia, *Bradyrhizobium*, diversity, phylogeny, *Vigna*, distribution

## Abstract

*Vigna minima* is a climbing annual plant widely distributed in barren wilderness, grass land, and shrub bush of China and other countries such as Japan. However, the rhizobia nodulating with this plant has never been systematically studied. In order to reveal the biodiversity of nodulating rhizobia symbiosis with *V. minima*, a total of 874 rhizobium isolates were obtained from root nodules of the plant spread in 11 sampling sites of Shandong Peninsula, China, and they were designated as 41 haplotypes in the genus *Bradyrhizobium* based upon *recA* sequence analyses. By multilocus sequence analysis (MLSA) of five housekeeping genes (*dnaK*, *glnII*, *gyrB*, *recA*, and *rpoB*), the 41 strains representing different *recA* haplotypes were classified into nine defined species and nine novel genospecies. *Bradyrhizobium elkanii*, *Bradyrhizobium ferriligni*, and *Bradyrhizobium pachyrhizi* were the predominant and universally distributed groups. The phylogeny of symbiotic genes of *nodC* and *nifH* showed similar topology and phylogenetic relationships, in which all the representative strains were classified into two clades grouped with strains nodulating with *Vigna* spp., demonstrating that *Vigna* spp. shared common nodulating groups in the natural environment. All the representative strains formed nodules with *V. minima* in a nodulation test performed in green house conditions. The correlation between *V. minima* nodulating rhizobia and soil characteristics analyzed by CANOCO indicates that available nitrogen, total nitrogen, and organic carbon in the soil samples were the main factors affecting the distribution of rhizobia isolated in this study. This study systematically uncovered the biodiversity and distribution characteristics of *V. minima* nodulating rhizobia for the first time, which provided novel information for the formation of the corresponding rhizobium community.

## Introduction

The genus *Vigna* is a member of the legume family, consisting of more than 100 species, which widely spread all over the world, mainly in warm temperate and tropical regions (Sakai et al., 2015). Species within the genus include essential and valuable crop plants such as *Vigna unguiculata* (L.) Walp. and *Vigna radiata* (L.) R. Wilczek and are cultivated mainly in Asian countries with approximately 11 million hectares (Ramírez et al., 2020). However, a number of wild species, such as *Vigna minima* (Roxb.), were also included in this genus that were found to possess some valuable phenotypic characteristics, such as high tolerance to severe conditions including high salinity, acidic or alkaline soil, drought and flooding, and other excellent phenotypes such as resistance to pest and disease and cross-compatibility (Chankaew et al., 2014; Tomooka et al., 2014; Sakai et al., 2015; Yoshida et al., 2016). Thus, the study and utilization of wild *Vigna* species are important not only for restoring the degenerated environmental conditions but also for obtaining some genetic resources with a potential for crop improvement (Ash et al., 2013).

Rhizobia are a kind of specific soil bacteria that could induce the formation of root and/or stem nodules and perform symbiotic biological fixation with legume hosts (Long, 1996; Masson-Boivin and Sachs, 2018; Bisseling and Geurts, 2020; Lindström and Mousavi, 2020). Currently, rhizobia encompass more than 180 species in 21 genera of two classes (*Alphaproteobacteria* and *Betaproteobacteria*), and the genera *Bradyrhizobium*, *Rhizobium*, *Mesorhizobium*, and *Ensifer* harbored the most common and the majority of the rhizobial species; furthermore, nodulation was thought to arose from *Bradyrhizobium* (Ormeño-Orrillo and Martínez-Romero, 2019; Chen et al., 2020).

As typical legume members, the cultured *Vigna* species could nodulate with diverse rhizobial species (Ramírez et al., 2020), but mainly the *Bradyrhizobium* species. The mung bean (*Vigna radiata* L.) plants mainly nodulate with *Bradyrhizobium japonicum*, *Bradyrhizobium liaoningense*, *Bradyrhizobium yuanmingense*, and *Bradyrhizobium elkanii* and rarely with members of the genera *Ensifer*, *Rhizobium*, and *Mesorhizobium* (Yang et al., 2008; Zhang et al., 2008; Risal et al., 2012; Hakim et al., 2018). The cowpea [*Vigna unguiculata* (L.ı) ıWalp.] plants mainly nodulate with *Bradyrhizobium pachyrhizi*, *Bradyrhizobium arachidis*, *B. yuanmingense*, and some novel *Bradyrhizobium* genomic species and rarely with members of *Rhizobium*, *Sinorhizobium*, and *Microvirga* (Zhang et al., 2007; Pule-Meulenberg et al., 2009; Radl et al., 2014; Tampakaki et al., 2017; Chidebe et al., 2018). The other two cultured *Vigna* species, *Vigna angularis* (Willd.) Ohwi & H. Ohashi (red bean) and *Vigna subterranea* L. Verdc. (bambara groundnut), were also mainly nodulated with diverse *Bradyrhizobium* species, and some strains were affiliated to *Sinorhizobium* (Han et al., 2009; Ibny et al., 2019). Due to the low economic value for the wild *Vigna* species, rhizobia nodulating with those plants are often overlooked and are rarely studied.

*Vigna minima* is an annual herb, twining plant, widely spread throughout the world such as China, Japan, India, Philippines, etc., which showed some important agronomic traits, such as higher protein content and nutritive value, salt tolerance, resistance to nematodes, and wider ecological amplitude (Gopinathan and Babu, 1986; Gopinathan et al., 1987). It widely spreads in barren soils such as grass land, roadside, shrub field, and sea beachside of China; however, no information is available about its rhizobia up to date. A study on the rhizobial diversity nodulating with *V. minima* could be a favor for the utilization of these wild genetic resources.

The aim of this study was to firstly systematically study the genetic diversity and phylogenetic relationships of *V. minima* nodulating rhizobia in Shandong Peninsula, China, through multilocus sequence analyses on housekeeping genes, and the relationship between biodiversity, community distribution, and environmental factors was also analyzed.

## Materials and Methods

### Soil and Nodule Sampling

**FIGURE 1:**
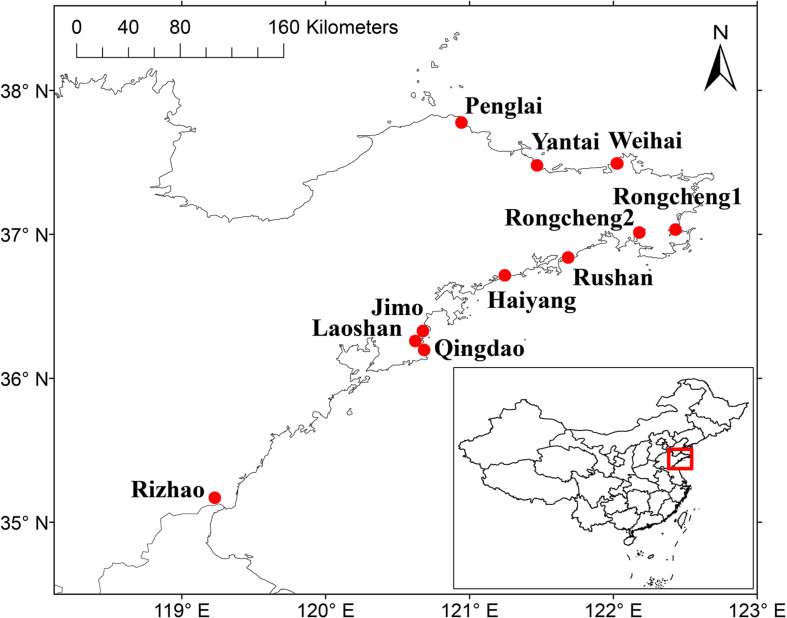
The sampling sites [filled circles (●)] in Shandong Peninsula, China. The geographic location of the Shandong Peninsula in China is shown in the inset. The two maps were created using the DIVA-GIS software (http://www.diva-gis.org), and the sampling sites were added according to GPS record data.

A total of 11 sampling sites were selected to collect nodules from *V. minima* and bulk soils around the plants, in Shandong Peninsula, China, in August 2018 (Figure 1). For each sampling site, more than 20 plants were randomly selected and uprooted; the nodules were carefully cut off from roots and were transferred into sealed plastic tubes half-filled with silica gel particles for preservation until isolation. At the same time, the rhizosphere and bulk soils (0–20-cm depth) were collected around each plant, were mixed into one sample for each sampling site, and were transferred to a laboratory where they were air dried and sieved by a 2-mm mesh screen.

### Rhizobial Isolation and Determination of Soil Physiochemical Characteristics

For rhizobial isolation, nodules collected from each sampling site were mixed, rehydrated, and surface sterilized. The nodules were crushed individually under aseptic condition, and the juice of each nodule was streaked onto the yeast mannitol agar (YMA: yeast extract 3.0 g, mannitol 10.0 g, K_2_HPO_4_ 0.5 g, MgSO_4_⋅6H_2_O 0.2 g, NaCl 0.1 g, agar 18 g, and distilled water 1,000 ml; pH 7.2) plates as described before (Li et al., 2016a; Zhang et al., 2020). All the inoculated YMA plates were incubated at 28°C for 1-4 weeks, and the plates were checked every 3 days for verifying the formation of bacterial colonies. One single colony was picked from each plate (nodule) and was purified by streaking repeatedly on the same medium until the colonies on the medium were homogeneous (Yao et al., 2014). Then, the purified cultures were suspended with sterilized 20% (*v*/*v*) glycerol and maintained at –80°C for long-term storage.

For determination of physiochemical traits, the air-dried, screened soil from each sampling site was analyzed according to the corresponding protocols (Li et al., 2016a). The pH values were determined through soil–water (1:2.5 *w*/*v*) suspensions by using a pH meter (Donohue, 1992). Available nitrogen (AN) content was determined through quantifying the alkali-hydrolyzed nitrogen (Shen et al., 2004). Available phosphorus (AP) content was determined through a colorimetry method by using a spectrophotometer (Westerman, 1990). Available potassium (AK) content was measured by the flame photometer methods with the corresponding protocol (Shen et al., 2004). Total nitrogen (TN) content was measured by titration methods (Page, 1982). And, the organic carbon (OC) content of the soil was measured by using the wet-oxidation method with K_2_Cr_2_O_7_-concentrated H_2_SO_4_ (Westerman, 1990).

### Phylogenetic Analyses of the Isolates

Genomic DNA of each isolate was extracted using TIANGEN genomic DNA extraction kit for bacteria (TIANGEN, China) and was used as the template to amplify *recA* sequences using a pair of primers of recA41F/640R (Vinuesa et al., 2005). The amplicons were directly sequenced using the Sanger methods at Beijing AuGCT DNA-SYN Biotechnology Co., Ltd. All the obtained sequences were aligned using ClustalW integrated in MEGA7.0 (Kumar et al., 2016), and the *recA* haplotypes were classified according to the instruction of DnaSP v5 (Librado and Rozas, 2009). A representative strain was randomly selected from each *recA* haplotype, and for all representative strains, the housekeeping genes including *dnaK*, *glnII*, *gyrB*, and *rpoB* were amplified by using primers of glnII12F/glnII689R (Yao et al., 2014), dnaK1171F/dnaK1748R, gyrB343F/gyrB1043R (Rivas et al., 2009), and rpoB454F/rpoB1346R (Martens et al., 2008) and the corresponding amplification methods. All the sequences obtained in this study were deposited in the GenBank database and were aligned to search for homologous reference sequences in GenBank. Multilocus sequence analysis (MLSA) was conducted by combining the sequences of five housekeeping genes (*dnaK*, *glnII*, *gyrB*, *recA*, and *rpoB*), and the sequence similarities between representative and reference strains were calculated using MEGA 7.0 (Kumar et al., 2016). A threshold of 97% sequence similarity was used to define genospecies as suggested previously (Cao et al., 2014; Li et al., 2016b).

### Symbiotic Characteristics of the Isolates

For each representative strain, the sequences of symbiotic genes of *nodC* and *nifH* were amplified and sequenced by using the primer pairs nodC540/1160 and nifH-F/nifH-R, respectively (Yao et al., 2014), with the corresponding PCR protocol. A nodulation test for each representative strain was performed under laboratory conditions using standard procedures as described previously. Seeds were surface sterilized, germinated, and transferred into a Leonard jar filled with sterilized vermiculite, which was irrigated with a low-nitrogen nutrient solution and inoculated with the desired rhizobial liquid inoculum (suspended in distilled water, 10^8^ cells/ml) (Vincent, 1970). Plants inoculated with distilled water were included as control. All the inoculated plants were grown at 24°C in an automated greenhouse with daylight illumination period of 12 h (Li et al., 2016a). Plants were harvested at 30 days post inoculation, and the plants with green leaves and pink round nodules were deemed as effective symbiosis with effective nodules, while the control plants remained with a small shoot size and without nodule on the root and showed yellow leaves.

### Diversity Evaluation and Correspondence Analyses

In this study, three common alpha ecological indices, including the Shannon–Wiener index (*H’*), the Simpson index (*D*), and the Pielou index (*J*), were calculated to explain the rhizobial species richness for a sample site, the species dominance, and the species evenness in a community, respectively, by using the Vegan package (version 1.17-4) and R (version 3.6.1) (Hill et al., 2003). The correspondence relationship between soil characteristics and rhizobial genospecies were evaluated by using the CANOCO 5.0 (ter Braak and Smilauer, 2012). Firstly, the rhizobial community was evaluated by detrended correspondence analysis (DCA); the length of the gradient (first axis) was 1.8, and thus, the RDA method was selected to perform the correspondence analyses.

## Results

### Soil Characteristics of the Sampling Sites

As shown in Table 1, the pH values of soil samples ranged from 6.39 in Qingdao to 7.45 in Yantai, which showed slight acidity to slight alkalinity. The content of the main mineral components in dry soils were (milligrams per kilogram) 16.7–120.1 for AN, 2.6–58.54 for AP, and 71.2–252.79 for AK and 0.03–0.15% for TN and 0.4–2.39% for OC.

**TABLE 1:** Relevant properties of soil samples and the distribution of different rhizobia genospecies.

**Properties**	**Sampling sites**										
	**Penglai**	**Yantai**	**Weihai**	**Rongcheng1**	**Rongcheng2**	**Rushan**	**Haiyang**	**Jimo**	**Laoshan**	**Qingdao**	**Rizhao**
GPS	N37.77 E120.93	N37.45 E121.49	N37.53 E122.06	N37.06 E122.43	N37.02 E122.23	N36.84 E121.68	N36.71 E121.25	N36.39 E120.68	N36.32 E120.63	N36.23 E120.67	N35.18 E119.27
**Physiochemical properties**											
PH	7.17	7.45	7.33	7.31	7.18	6.80	7.84	6.70	6.96	6.39	7.37
AN (mg kg^–1^)	31.50	23.10	94.70	17.20	16.70	36.40	51.50	120.10	52.40	84.70	90.53
AK (mg kg^–1^)	236.44	71.12	252.79	197.8	177.4	201.20	217.84	213.77	200.20	167.13	206.79
AP (mg kg^–1^)	10.36	7.14	58.54	9.08	2.60	5.02	5.50	5.46	7.06	6.78	8.20
OC (%)	1.22	0.54	0.40	0.44	2.39	0.91	0.71	0.76	0.80	0.58	0.47
TN (%)	0.15	0.08	0.05	0.03	0.15	0.07	0.09	0.08	0.10	0.09	0.06
Fertility level (N/K/P)^*a*^	5/1/3	6/4/4	3/1/1	6/2/4	6/2/6	5/1/4	5/1/4	2/1/4	5/1/4	4/2/4	3/1/4
**Rhizobial distribution**											
*B. yuanmingense*	0	0	0	0	0	0	11	0	1	3	0
*Bradyrhizobium* sp. I	0	0	1	0	0	0	0	0	0	0	0
*Bradyrhizobium* sp. II	0	11	7	3	1	0	1	0	6	2	0
*Bradyrhizobium* sp. III	0	0	0	1	1	0	0	0	0	0	0
*B. daqingense*	0	1	0	0	0	0	0	0	1	0	0
*B. liaoningense*	0	2	0	1	0	0	10	0	0	0	0
*Bradyrhizobium* sp. IV	0	0	2	0	3	0	0	8	1	0	0
*B. arachidis*	0	0	0	1	0	3	0	0	0	0	0
*B. ottawaense*	8	0	1	0	6	0	0	0	1	0	0
*Bradyrhizobium* sp. V	0	0	0	0	0	3	0	0	0	0	0
*Bradyrhizobium* sp. VI	1	0	0	0	0	0	0	0	0	0	0
*B. lablabi*	0	0	0	0	0	0	0	0	3	0	0
*Bradyrhizobium* sp. VII	0	9	0	4	6	2	4	0	1	0	0
*Bradyrhizobium* sp. VIII	0	0	0	0	0	0	0	0	2	0	0
*Bradyrhizobium* sp. IX	0	0	1	0	0	0	0	0	0	0	0
*B. elkanii*	11	73	53	47	46	38	5	44	32	26	58
*B. ferriligni*	29	25	4	25	34	17	25	16	32	19	0
*B. pachyrhizi*	1	6	2	6	22	12	4	6	17	3	2
Total strain number	50	127	71	88	119	75	60	74	97	53	60
Shannon–Wiener	1.10	1.29	0.99	1.28	1.51	1.33	1.61	1.08	1.63	1.17	0.15
Simpson	0.59	0.62	0.43	0.63	0.73	0.66	0.75	0.58	0.75	0.62	0.06
Inverse Simpson	2.43	2.6	1.75	2.67	3.69	2.96	3.98	2.39	3.94	2.65	1.07
Pielou index	0.68	0.66	0.48	0.62	0.73	0.74	0.83	0.78	0.68	0.72	0.21

*^*a*^According to the China national standard, level 1 = very rich, 2 = rich, 3 = moderate, 4 = poor, 5 = very poor, 6 = extremely poor (http://www.soil17.com/news more/1663.html).*

### Rhizobial Isolation and Selection of Representative Strains

A total of 874 isolates were obtained from 11 sampling sites in this study; the numbers of isolates obtained from the sites varied from 50 in Penglai to 127 in Yantai (Table 1 and Supplementary Tables 1, 2). Forty-one *recA* haplotypes, in which nine covered only one strain and H30 covered 238 strains, were classified according to the *recA* sequence analysis, and one randomly selected representative for each haplotype was used for the subsequent phylogenetic analyses (Supplementary Table 1). According to the phylogenetic tree based on *recA* sequences, all the representative strains were classified into the genus of *Bradyrhizobium*.

### Phylogeny and Species Affiliation of the Rhizobial Isolates Based on MLSA

**FIGURE 2:**
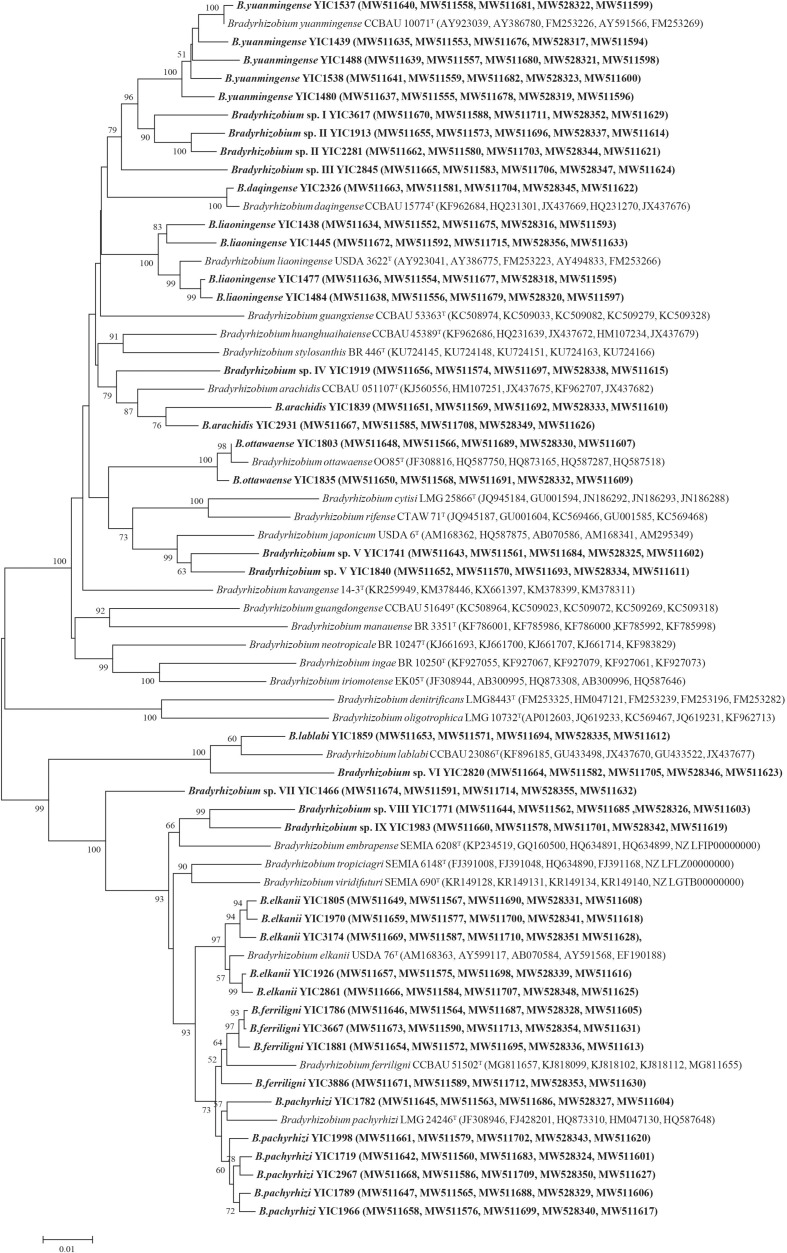
Multilocus sequence analysis (MLSA) phylogenetic tree based on concatenated sequence of *dnaK* (451 bp), *glnII* (558 bp), *gyrB* (605 bp), *recA* (455 bp), and *rpoB* (761 bp). Taxa and GenBank accession numbers in boldface were isolated and determined in this study. The tree was constructed by the neighbor-joining method using MEGA 7.0. Bootstrap values larger than 50% are shown at the nodes. The scale bar represents 1% substitution of the nucleotide.

To determine the accurate taxonomy positions, sequences of the house keeping genes *atpD*, *dnaK*, *glnII*, *gyrB*, and *rpoB* were obtained from all the 41 representative strains that have been deposited in the GenBank database and the accession numbers were listed in Supplementary Table 3. In the phylogenetic tree of MLSA (Figure 2) based on the concatenated sequences of the five housekeeping genes (*dnaK*, *glnII*, *gyrB*, *recA*, and *rpoB*), the 41 representative strains were classified as 18 genospecies including nine validly published species and nine candidate novel genospecies (Figure 2, Table 1, and Supplementary Table 4). Among the nine defined species, *B. elkanii* (*recA* haplotypes H27–H31 with 433 isolates), *B. ferriligni* (H32–H35 with 226 isolates), and *B. pachyrhizi* (H36–H41 with 81 isolates) were dominant. The remaining species *B. yuanmingense*, *B. daqingense*, *B. liaoningense*, *B. arachidis*, *B. ottawaense*, and *B. lablabi* were site specific covering 2 to 16 isolates in one to five haplotypes. The novel genospecies were named as *Bradyrhizobium* sp. I, through sp. IX, in which sp. II and sp. VII covered 31 and 26 isolates from seven and six sampling sites, respectively, while the remaining harbored 1 to 14 isolates. These novel genospecies were most related to *B. yuanmingense* CCBAU 10071^*T*^, *B. arachidis* CCBAU 051107^*T*^, *B. japonicum* USDA 6^*T*^, *B. lablabi* CCBAU 23086^*T*^, *Bradyrhizobium ferriligni* CCBAU 51502^*T*^, or *B. embrapense* SEMIA 6208^*T*^ with similarities less than 96.8% in MLSA.

### Distribution and Biodiversity of Rhizobia in the Sampling Sites

**FIGURE 3:**
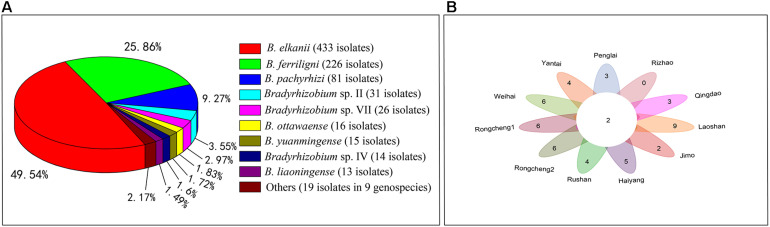
The statistical data of *V. minima*-nodulating rhizobia isolated in this study. **(A)** The diagram showing the relative percentage abundances of the genospecies nodulating with *V. minima*. The genospecies ratio accounting more than 1% was provided. **(B)** All the sampling sites commonly shared two genospecies (the number in the center circle) of *B. elkanii* and *B. pachyrhizi*. The numbers in each part of the flower indicate the remaining genospecies number subtracted from the commonly shared genospecies.

Among the 18 genospecies classified in the rhizobia of *V. minima*, the predominant genospecies (>5%) were *B. elkanii* (433 isolates), *B. ferriligni* (226 isolates), and *B. pachyrhizi* (81 isolates), accounting for 49.49%, 25.83%, and 9.26% of total isolates (Figure 3A and Table 1), while only one isolate was detected in each of the genospecies *Bradyrhizobium* sp. I, sp.VI, and sp. IX (Table 1 and Supplementary Table 2). The genospecies number distributed in the sampling sites varied from 2 in Rizhao to 11 in Laoshan. Two genospecies of *B. elkanii* and *B. pachyrhizi* were distributed in all of the sampling sites, followed by *B. ferriligni*, which was distributed in 10 of the 11 sampling sites (Figure 3B and Table 1). The highest diversity index of Shannon–Weiner (*H′*, 1.63) was observed in Laoshan, followed by that in Haiyang (1.61) and Rongcheng2 (1.51), and the lowest index value (0.15) was observed in Rizhao where only two genospecies were identified. The highest Simpson’s index (*D*) observed varied from 0.06 in Rizhao to 0.75 in Haiyang. The evenness index value of Pielou (*J*) varied between 0.21 in Rizhao and 0.83 in Haiyang (Table 1).

### Phylogeny of the Isolates Based on Symbiotic Genes (*nodC* and *nifH*)

**FIGURE 4:**
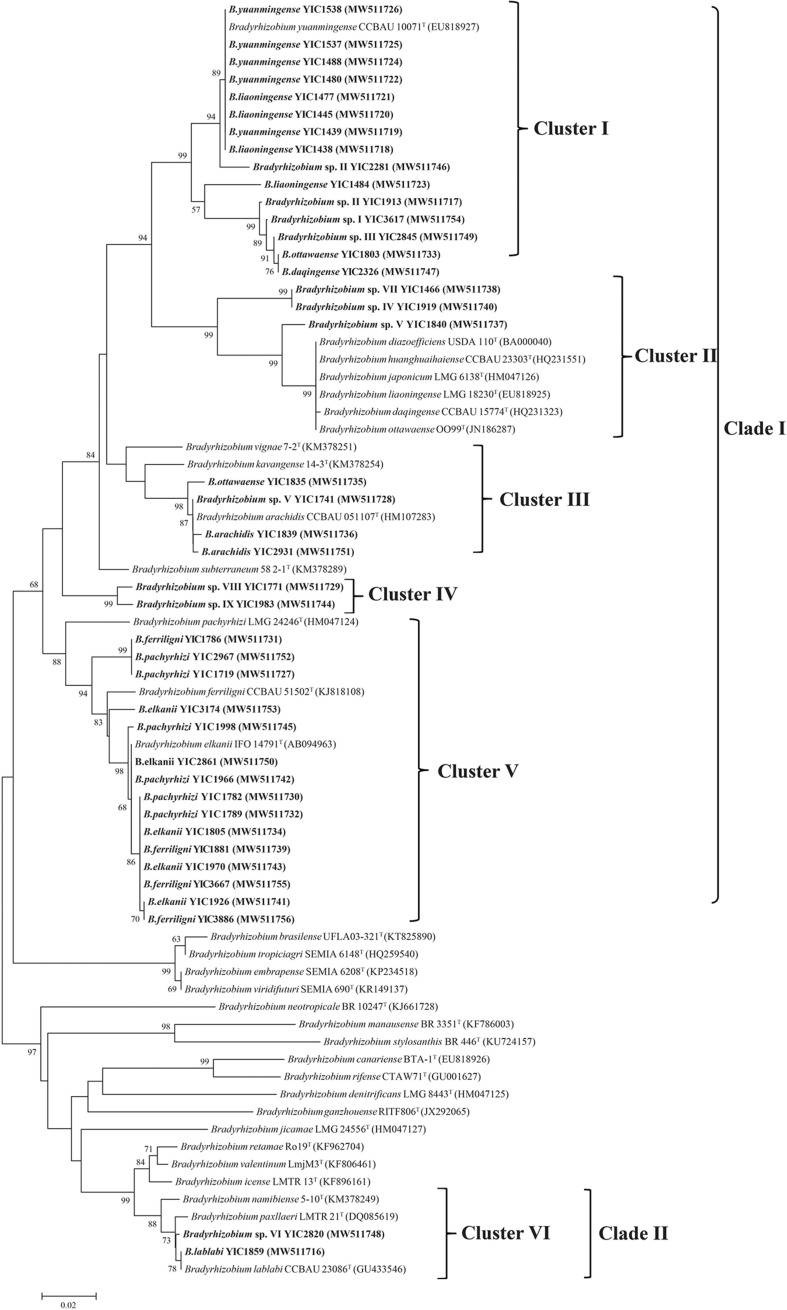
Neighbor-joining tree based on *nifH* sequences of representative strains isolated in this study and reference strains. The tree was reconstructed using MEGA 7.0, and bootstrap values greater than 50% are shown at the nodes. The scale bar represents 2% nucleotide substitutions.

The *nifH* sequence of all the representative strains was amplified successfully; however, the *nodC* sequencing of *Bradyrhizobium* sp. II YIC2281 and *B. daqingense* YIC2326 failed. In the phylogenetic tree of *nifH* and *nodC*, the topology and relationship of representative strains were very similar. They both formed two clades and five clusters (Figure 4 and Supplementary Figure 6). Clade A consisted of four clusters, which encompassed most (39/41, accounting 95% of the total representative strains) of the representative strains. Clade B only consisted of a single cluster (cluster V for *nifH* or cluster E for *nodC*) with two strains of *Bradyrhizobium* sp. VI YIC2820 and *B. lablabi* YIC1859 (Figure 4 and Supplementary Figure 6). All the representative strains could nodulate with the original host *V. minima* in a nodulation test performed in greenhouse.

### Correlation of Soil Properties and Distribution of *V. minima* Rhizobia

**FIGURE 5:**
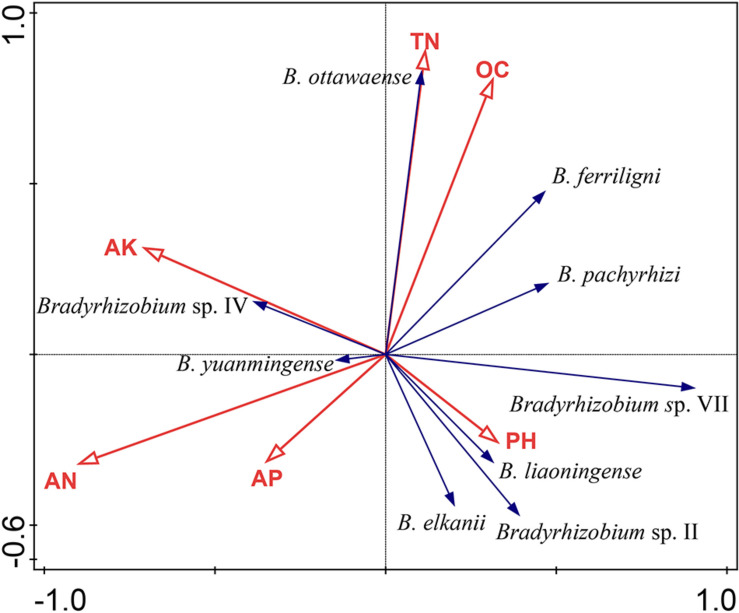
Corresponding analyses between the genospecies and the soil physiochemical characteristics calculated by CANOCO. Genospecies accounting more than 1% was selected to perform the calculation. AN, available N; AP, available P; AK, available K; OC, organic carbon; TN, total N.

In the DCA test, the length of the gradient (first axis) was 1.8, which demonstrates that the linear method was suitable to our analyses, and the RDA result is presented in Figure 5, in which axis 1 and axis 2 explained 38.29% of the variation of species–soil characteristic correlation. According to the length of the soil characteristics, AN, TN, and OC were the main factors affecting the distribution of *V. minima* genospecies (Figure 5), as they explained 42.7% of the total variant and contributed 64.5% of the distribution of the genospecies. Furthermore, the effect of AN showed to be the most obvious (*P* = 0.026). The predominant groups *B. ferriligni* and *B. pachyrhizi* were negatively correlated with AN and AP, but positively correlated with OC. TN positively affected the distribution of *B. ottawaense* and *B. ferriligni*. The distribution of *B. ottawaense* was also positively affected by OC. AK positively affected the distribution of *B. yuanminense* and *Bradyrhizobium* sp. IV, but negatively affected the distribution of *Bradyrhizobium* sp. VII, *B. liaoningense*, *Bradyrhizobium* sp. II, and *B. elkanii* (Figure 5).

## Discussion

Although it is an endemic plant in the seashore of Shandong, *V. minima* and its rhizobia have been overlooked up to now. In this study, a total of 874 rhizobial isolates were isolated from root nodules of *V. minima* grown in 11 sampling sites. In previous studies, we found that *recA* sequence showed a higher resolution in classification of haplotypes than other housekeeping genes such as *atpD*, *glnII*, and *rpoB* (data not shown). In this study, *recA* also showed more robust than any other housekeeping genes in classifying haplotypes, for it generated the highest number of different haplotypes and the lowest similarity values between different haplotypes (Supplementary Figures 1–5 and Supplementary Tables 4, 5). Thus, *recA* is superior to other markers and could be widely used in defining rhizobial haplotypes. The definition of 41 haplotypes in *Bradyrhizobium* based on *recA* sequences among these isolates primitively evidenced their great genetic diversity and further confirmed the *recA* sequence analysis as an effective screening method to group the rhizobial isolates, identify the genus, and select the representative strains (Supplementary Tables 1, 2) for further phylogenetic study (Li et al., 2016a, b; Shao et al., 2020; Zhang et al., 2020). In MLSA of the five housekeeping genes (*glnII*, *dnaK*, *gyrB*, *rpoB*, and *recA*), the identification of 18 *Bradyrhizobium* genospecies among the isolates revealed that *V. minima* only nodulated with *Bradyrhizobium* in the studied sites that is consistent with the cultured *Vigna* species (Yang et al., 2008; Han et al., 2009; Hakim et al., 2018; Ibny et al., 2019). However, the detection of 18 genospecies evidenced the possibility that *V. minima* might not be very stringent for the genomic background of its microsymbionts. Also, these results implied that the *Bradyrhizobium* communities might have co-evolved or diversified together with *V. minima* for a long time in Shandong.

Among the nine defined species identified in the present study, *B. arachidis, B. elkanii, B. liaoningense, B. pachyrhizi*, and *B. yuanmingense* have been reported as microsymbionts for the four cultured *Vigna* species mentioned in introduction. The remaining four species and the nine novel genospecies were new records for microsymbionts of *Vigna.* These results improved our knowledge about the biodiversity of *Vigna* rhizobia and enlarged the host spectrum of *B. daqingense* (Wang et al., 2013), *B. ferriligni* (Yao et al., 2015), *B. lablabi* (Chang et al., 2011), and *B. ottawaense* (Yu et al., 2014). On the other hand, the dominant groups of *Bradyrhizobium japonicum* and *Ensifer* (*Sinorhizobium*) and the minor groups of *Rhizobium*, *Mesorhizobium*, and *Microvirga* isolated from other *Vigna* species were not isolated in the present study. It is not clear whether the differences among the rhizobial communities associated with different *Vigna* species were results of the host–rhizobial specificity or caused by the soil/environmental conditions.

In the present study, nine dominant genospecies (>1% of the total isolates) were defined, including *B. elkanii* (49.49%), *B. ferriligni* (25.83%), *B. pachyrhizi* (9.26%), *Bradyrhizobium* sp. II (3.54%), *Bradyrhizobium* sp. VII (2.97%), *B. ottawaense* (1.83%), *B. yuanmingense* (1.71%), *Bradyrhizobium* sp. IV (1.6%), and *B. liaoningense* (1.49%) (Figure 3A). The predominance of *B. elkanii* and *B. pachyrhizi* was in accordance with the previous reports for rhizobia of other *Vigna* species such as *V. radiata*, *V. unguiculata*, *Vigna subterranea*, and *V. angularis* (Zhang et al., 2007; Yang et al., 2008; Han et al., 2009; Ibny et al., 2019; Puozaa et al., 2019). The super dominance and ubiquitous distribution in the 11 sampling sites of *B. elkanii*, *B. pachyrhizi*, and *B. ferriligni* (Figure 3 and Table 1) demonstrated them as the most adapted or affinity symbionts of *V. minima* in the study area. Furthermore, in the phylogenetic tree, these three species form a clade with high similarities (Figure 2), implying the similar genomic backgrounds among them that may explain their dominance in this study from a genomic insight. In other words, these three dominant rhizobial species supported well the colonization/spread of *V. minima* in the beachside of Shandong Peninsula, while the existence of minor rhizobial groups offered the host more choices for symbionts when variation occurs in the natural environment.

Comparing with the great diversity of species, the diversity of symbiosis genes among the *V. minima* rhizobia was less, since the same five clusters were defined among the 41 representative strains in both the *nifH* and *nodC* phylogenies (Figure 4 and Supplementary Figure 6), except that *nodC* gene was not successfully sequenced for the strains *Bradyrhizobium* sp. II YIC2281 and *B. daqingense* YIC2326. The symbiosis genes in five of the six clusters were identical or similar to those of the strains nodulating with *Lespedeza*, peanut, soybean, and *Lablab*, except the cluster IV that was an independent lineage. These results demonstrated that the symbiosis genes of *V. minima* may have different origins or have diversified in the study area, and *V. minima* might share their microsymbionts with the legumes mentioned above. Among the symbiosis gene clusters, clusters I and V for *nifH* gene (clusters A and E for *nodC*) were dominant, and each encompassed 15 of the 42 representative strains. The 15 test strains in cluster V included all the representative strains to *B. elkanii*, *B. ferriligni*, and *B. pachyrhizi*, as well as the type strains for these three species, while the 15 test strains in cluster I of the *nifH* phylogenetic tree (Figure 4) contained all the representative strains for *B. liaoningense*; *Bradyrhizobium* sp. I, sp. II, and sp. III; *B. daqingense*; and a representative for *B. ottawaense*, as well as the type strain of *B. yuanmingense* CCBAU 10071^*T*^. These intermingle cases of different rhizobial species in the phylogenetic trees of symbiosis genes have been reported in many previous studies and were believed as evidence of lateral transfer of symbiosis genes among the related species. Thus, *V. minima* not only selects the genomic backgrounds but also the symbiosis genes of the nodulating rhizobia. The studies on both housekeeping and symbiotic genes of *Vigna* nodulating rhizobia further demonstrate the plants in various *Vigna* species could form symbiosis with rhizobia in distinct species harboring rather diverse symbiosis genes in the natural environment, just like the case of soybean (Zhang et al., 2012). In this study, all the representative strains could form effective nodules with their original host in the nodulation test, and the reason for the failure to amplify *nodC* sequences of strain YIC2281 and YIC2326 is unknown.

Although we could not verify the real causes for the composition differences among the rhizobial communities associated with distinct *Vigna* species as mentioned above, sampling the rhizobia from same plants grown in different sites in the present study offered us an adequate model to analyze the effects of soil traits on rhizobial community composition. Firstly, the diversity index of rhizobial community varied (0.15 to 1.63) for different sampling sites in our study (Table 1), which is an overall evidence for the effects of soil traits on rhizobial community. Secondly, the species numbers in the sampling sites varied between 2 and 11 (Figure 3B), indicating many rhizobial (geno)species have site specific distribution in this study. So, our results evidenced that soil traits affected the distribution and community composition, which is consistent with the observations in the previous studies (Zhang et al., 2012; Cao et al., 2014). In the present study, the AN content in most of the soils, except that in Jimo, was moderately to extremely poor (Table 1). Except Weihai and Penglai, the AP contents in the soils were also poor to very poor (Table 1). Thus, the *V. minima*-grown soils were barren, and the nodules induced by rhizobia may be the main nitrogen source for the plant. However, except Yantai, the AK content in all the soil was rich to very rich, which might be mainly released from the process of decaying plant straw (Liu et al., 2020). Comparing the soil traits and the distribution of rhizobial (geno)species, it looks like the sampling sites with lower AN content have higher diversity indexes, which is consistent with the results of a correspondence analysis (Figure 5) that the AN content was negatively correlated with the distribution of most of the genospecies isolated in this study, similar to the observations for soybean rhizobial community (Zhang et al., 2012). Different from some previous studies (Zhang et al., 2012), the pH value of soils did not strongly correlate with the distribution of rhizobial (geno)species in this study, which might be explained by the fact that the pH values of the soil samples involved in this study only varied from slightly acidic to slightly alkaline.

## Conclusion

In conclusion, a unique *V. minima* nodulating rhizobial community structure from Shandong Peninsula, China, was systematically uncovered in this study. The plant-nodulating bacteria belong to 18 *Bradyrhizobium* genospecies including nine validly published and nine novel genospecies. The predominant groups were *B. elkanii*, *B. ferriligni*, and *B. pachyrhizi*. The symbiosis genes *nifH* and *nodC* of the representative strains were grouped into five clusters related to the reference strains nodulating with *Lablab*, *Lespedeza*, peanut, and soybean. Therefore, *V. minima* selects both the genomic background and symbiotic genes of the nodulating rhizobia. The present study is the first systematic assessment of the wildly unattended plant *V. minima*-nodulating rhizobia, which enlarged the diversity of *Vigna-*nodulating rhizobia and provided an insight of the co-evolution between this legume and its rhizobia.

## Data Availability Statement

The data used in this study have been deposited in the GenBank database and the accession numbers are listed in Supplementary Table 3.

## Author Contributions

YL, ZZ, and WD conceived and designed the experiments. GL, XL, WL, ZZ, and XC performed the experiments. GL and YL performed the analysis and wrote the manuscript. E-TW revised the manuscript. All authors have read and agreed to publish the manuscript.

## Conflict of Interest

The authors declare that the research was conducted in the absence of any commercial or financial relationships that could be construed as a potential conflict of interest.
