# Supplementary material for: Biodiversity and Geographic Distribution of Rhizobia Nodulating With Vigna minima
Source: Front Microbiol. 2021 May 4;12:665839. doi: 10.3389/fmicb.2021.665839 (PMC8129581; doi:10.3389/fmicb.2021.665839)
Supplement: Supplementary file 1 [file Data_Sheet_1.docx]

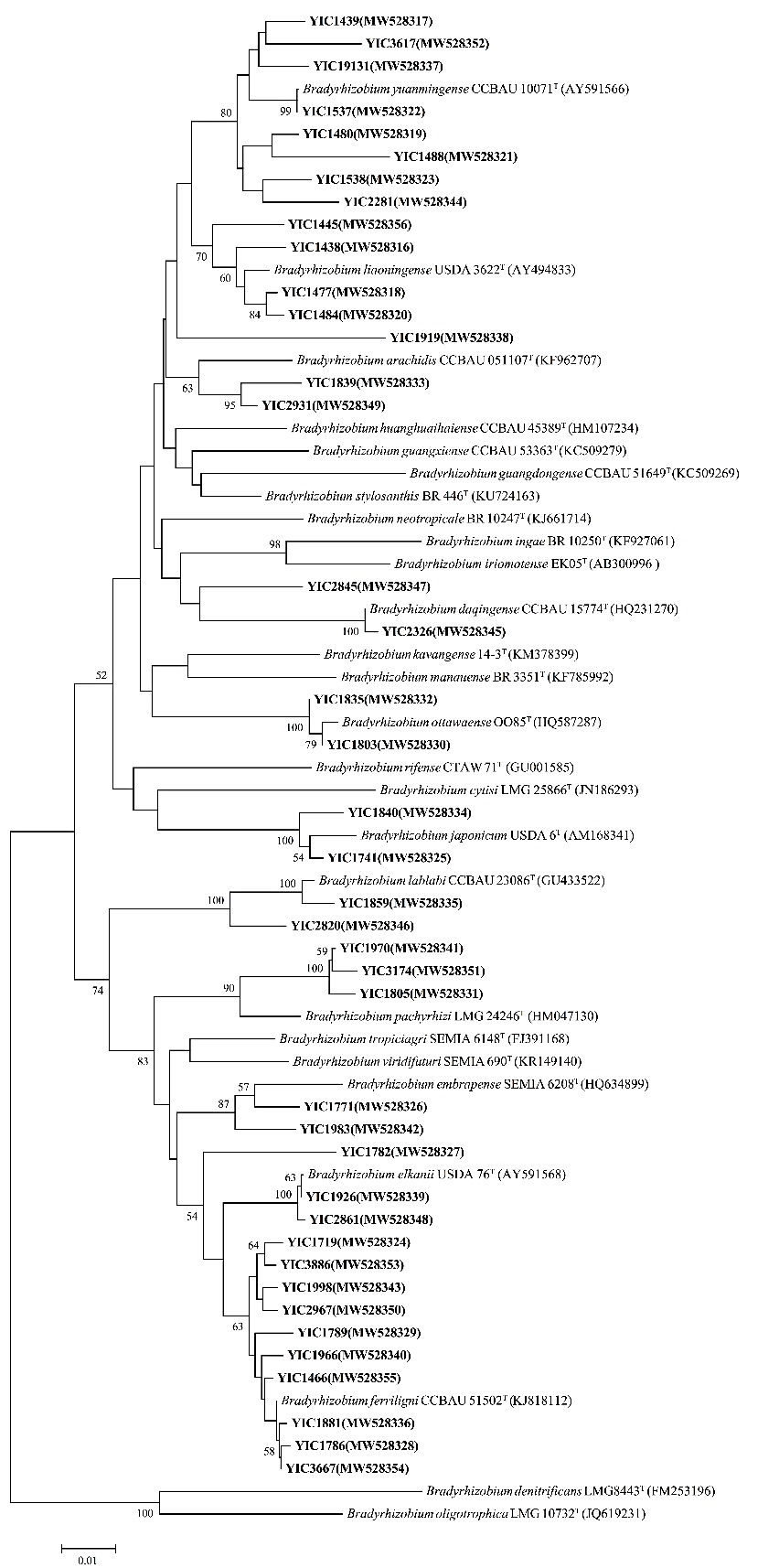


**Fig. S1.** Phylogenetic tree of *recA* sequences showing the relationships between the representative strains isolated in this study (in boldface) and the related species. The Neighbor-joining tree was reconstructed using MEGA 7.0, and Kimura 2-parameter model was selected as the nucleotide substitution model. Bootstrap confidence levels of ≥ 50% are indicated at the internodes. The bar indicates 1% nucleotide divergence.


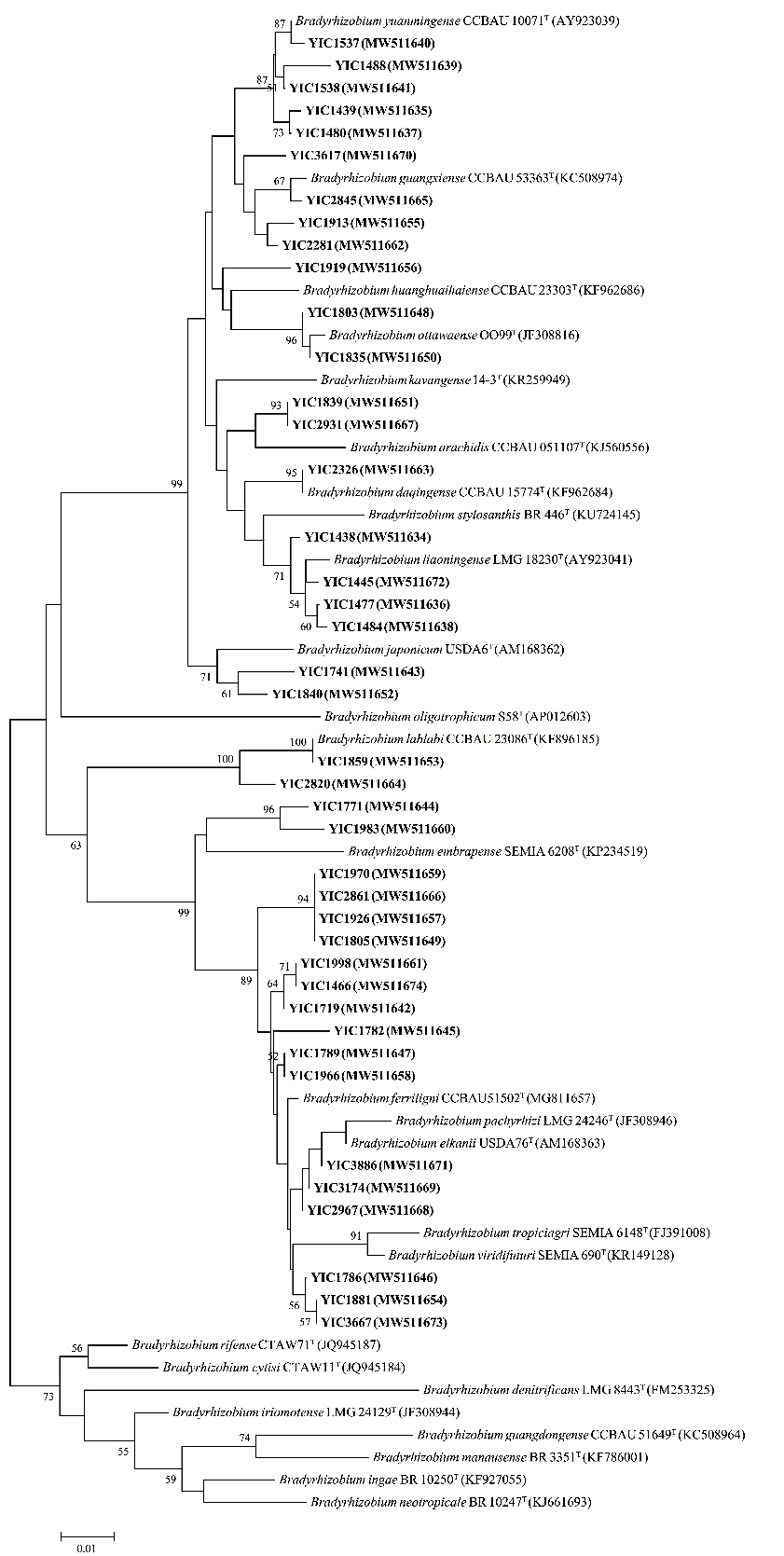


**Fig. S2.** Phylogenetic tree of *dnaK* sequences showing the relationships between the representative strains isolated in this study (in boldface) and the related species. The Neighbor-joining tree was reconstructed using MEGA 7.0, and Kimura 2-parameter model was selected as the nucleotide substitution model. Bootstrap confidence levels of ≥ 50% are indicated at the internodes. The bar indicates 1% nucleotide divergence.


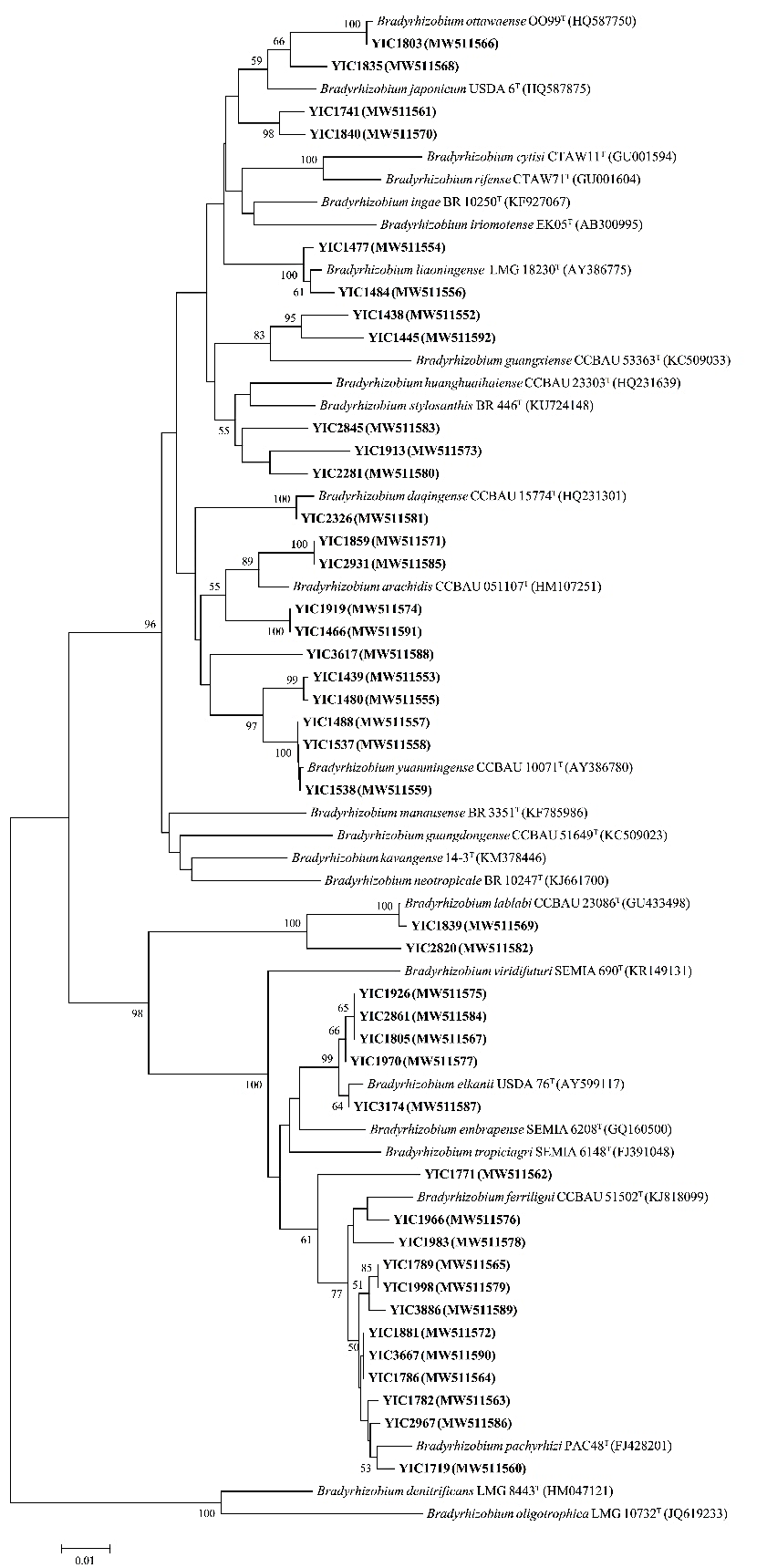


**Fig. S3**. Phylogenetic tree of *glnII* sequences showing the relationships between the representative strains isolated in this study (in boldface) and the related species. The Neighbor-joining tree was reconstructed using MEGA 7.0, and Kimura 2-parameter model was selected as the nucleotide substitution model. Bootstrap confidence levels of ≥ 50% are indicated at the internodes. The bar indicates 1% nucleotide divergence.


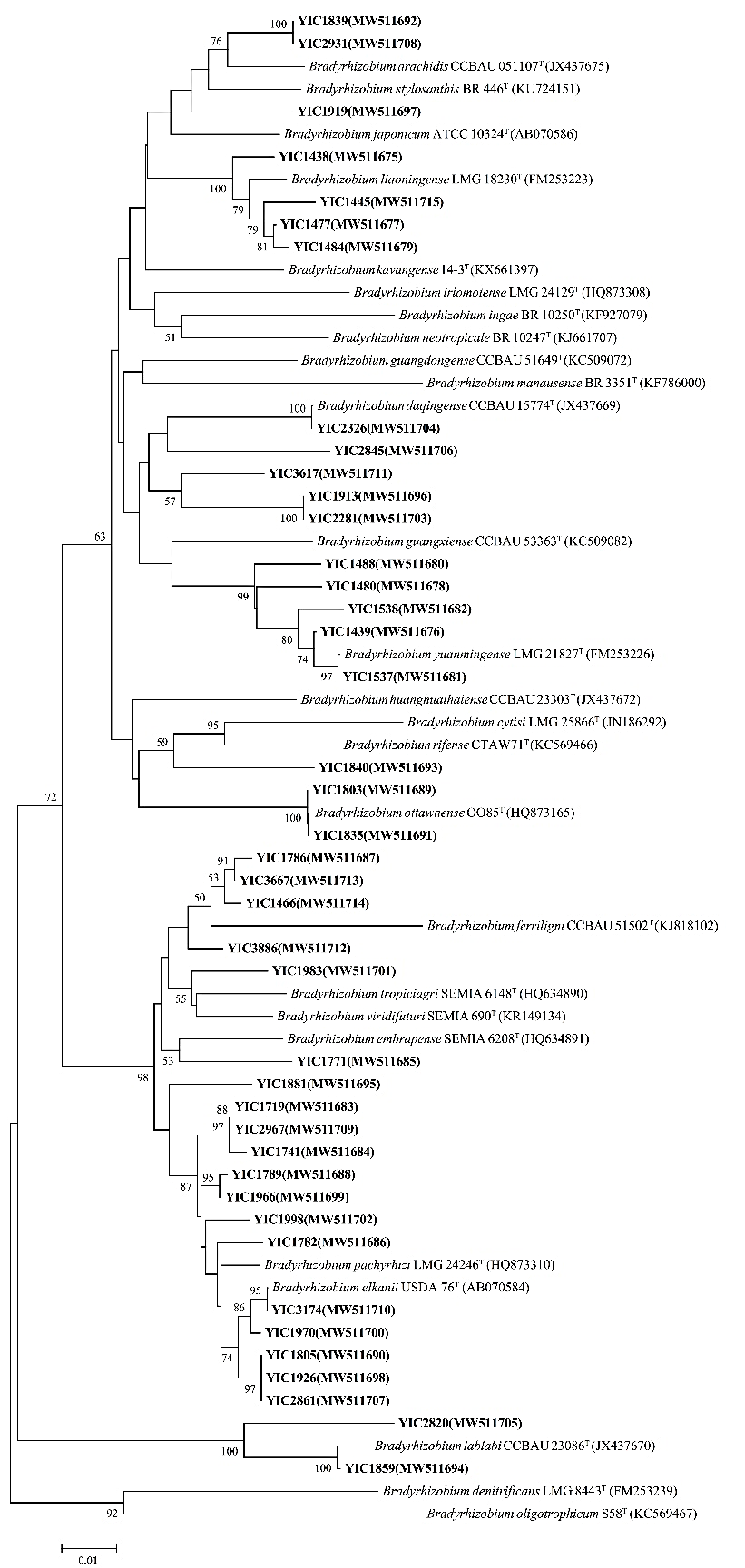


**Fig. S4**. Phylogenetic tree of *gyrB* sequences showing the relationships between the representative strains isolated in this study (in boldface) and the related species. The Neighbor-joining tree was reconstructed using MEGA 7.0, and Kimura 2-parameter model was selected as the nucleotide substitution model. Bootstrap confidence levels of ≥ 50% are indicated at the internodes. The bar indicates 1% nucleotide divergence.


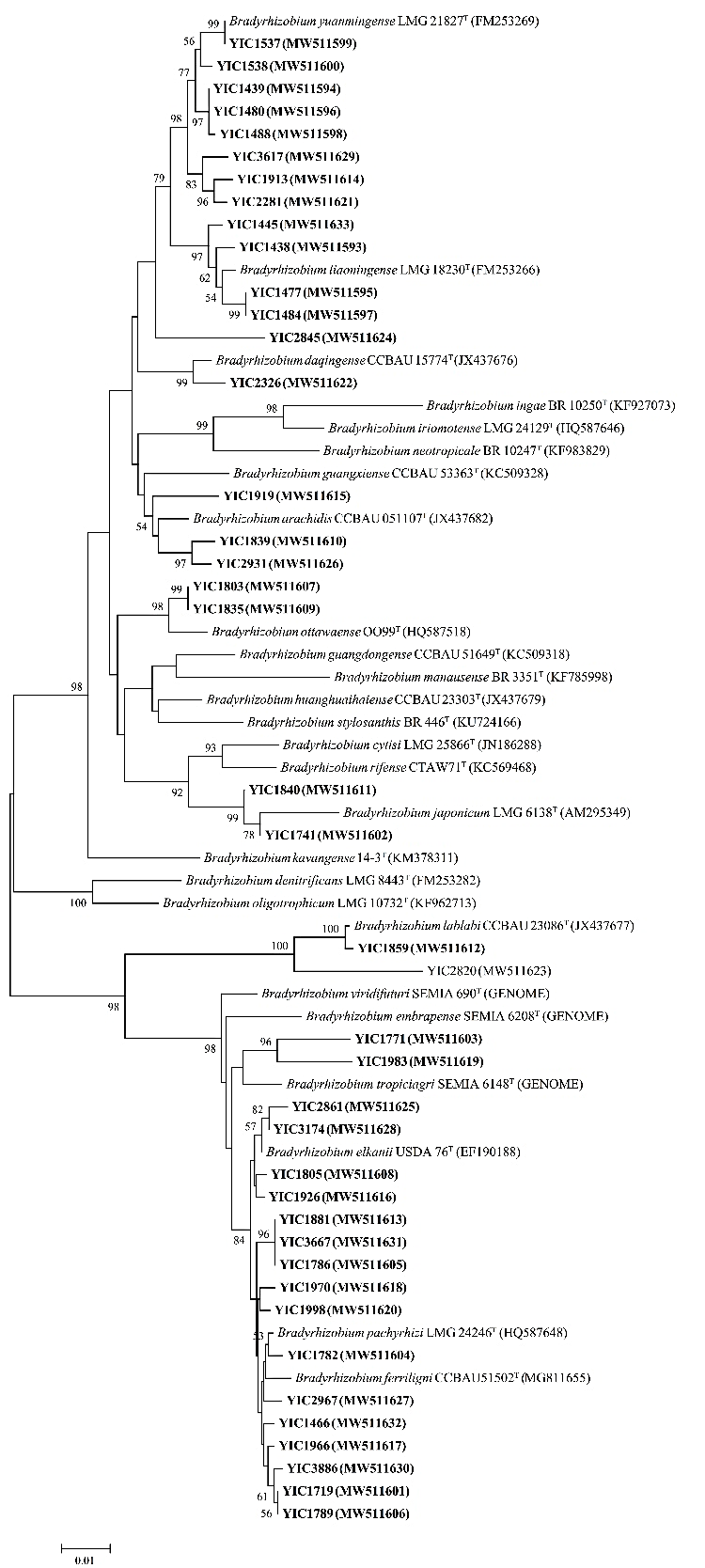


**Fig. S5**. Phylogenetic tree of *rpoB* sequences showing the relationships between the representative strains isolated in this study (in boldface) and the related species. The Neighbor-joining tree was reconstructed using MEGA 7.0, and Kimura 2-parameter model was selected as the nucleotide substitution model. Bootstrap confidence levels of ≥ 50% are indicated at the internodes. The bar indicates 1% nucleotide divergence.


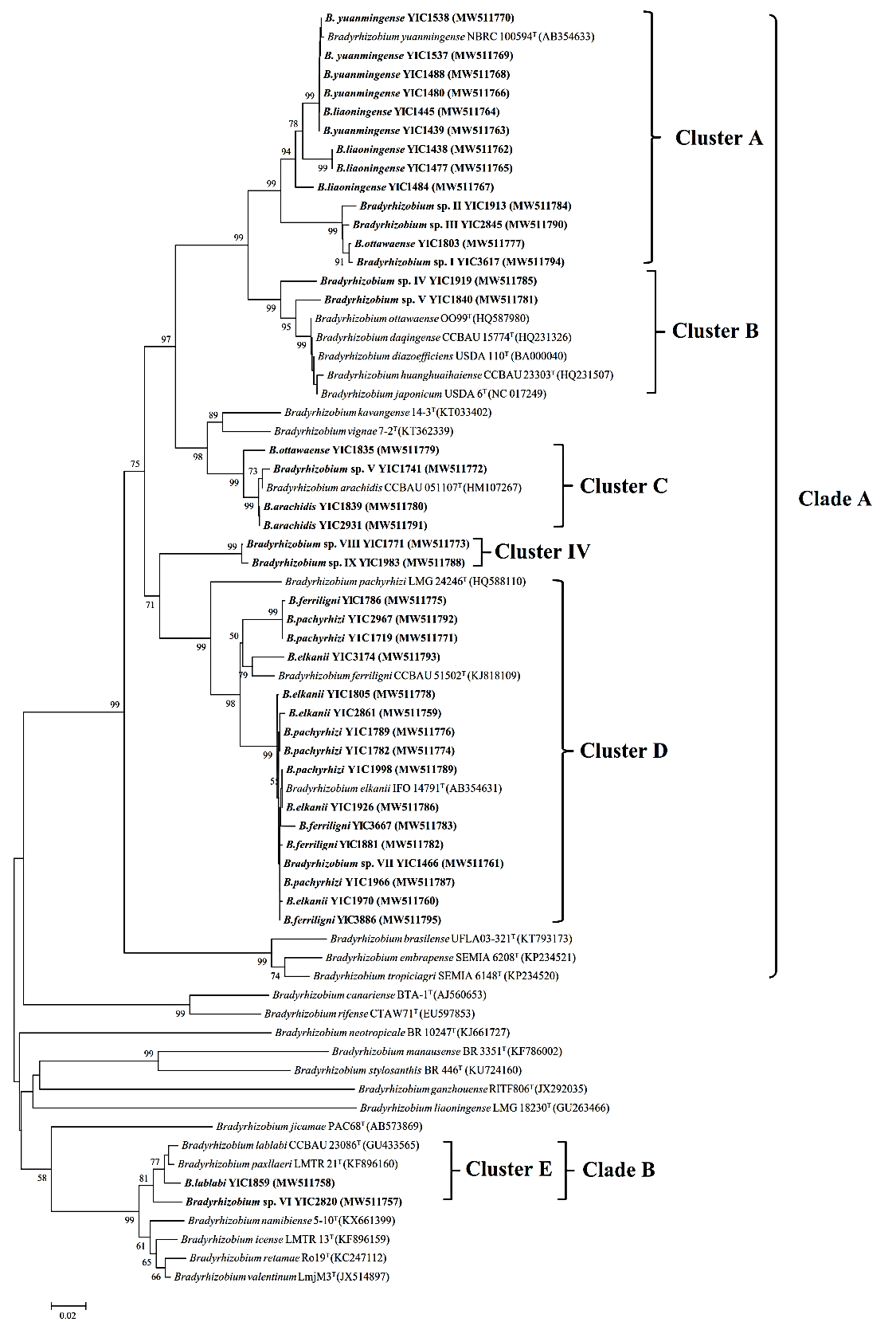


**Fig. S6**. Phylogenetic tree of *nodC* sequences showing the relationships between the representative strains isolated in this study (in boldface) and the related species. The Neighbor-joining tree was reconstructed using MEGA 7.0, and Kimura 2-parameter model was selected as the nucleotide substitution model. Bootstrap confidence levels of ≥ 50% are indicated at the internodes. The bar indicates 1% nucleotide divergence.

**Table S1.** The list of *recA* haplotypes classification of *V. minima* nodulating rhizobia isolated in this study.

| **Classification** | **Haplotypes No.** | **Representative strain** | **Number of Isolates** | **Distribution (number of strains) in sampling site** |
| --- | --- | --- | --- | --- |
| *B. yuanmingense*  (15 isolates) | H1 | YIC1537 | 6 | Haiyang (5), Qingdao (1) |
|  | H2 | YIC1439 | 1 | Haiyang (1) |
|  | H3 | YIC1488 | 3 | Haiyang (3) |
|  | H4 | YIC1538 | 3 | Haiyang (1), Qingdao (2) |
|  | H5 | YIC1480 | 2 | Haiyang (1), Laoshan (1) |
| *Bradyrhizobium* sp. I | H6 | YIC3617 | 1 | Weihai (1) |
| *Bradyrhizobium* sp. II  (31 isolates) | H7 | YIC1913 | 30 | Weihai (7), Rongcheng 1 (3), Rongcheng 2 (1), Haiyang (1), Laoshan (6), Qingdao (2), Yantai (10) |
|  | H8 | YIC2281 | 1 | Yantai(1) |
| *Bradyrhizobium* sp. III | H9 | YIC2845 | 2 | Rongcheng 1 (1), Rongcheng 2 (1) |
| *B. daqingense* | H10 | YIC2326 | 2 | Laoshan (1), Yantai (1) |
| *B. liaoningense*  (13 isolates) | H11 | YIC1438 | 2 | Haiyang (1), Yantai (1) |
|  | H12 | YIC1445 | 3 | Haiyang (1), Rongcheng 1 (1), Yantai (1) |
|  | H13 | YIC1477 | 1 | Haiyang (1) |
|  | H14 | YIC1484 | 7 | Haiyang (7)M |
| *Bradyrhizobium* sp. IV | H15 | YIC1919 | 14 | Jimo (8), Laoshan (1), Weihai (2), Rongcheg 2 (3) |
| *B. arachidis* (4 isolates) | H16 | YIC1839 | 3 | Rushan (3) |
|  | H17 | YIC2931 | 1 | Rongcheng 1 (1) |
| *B. ottawaense*  (16 isolates) | H18 | YIC1803 | 14 | Rongcheng 2 (4), Laoshan (1), Penglai (8), Weihai (1) |
|  | H19 | YIC1835 | 2 | Rongcheng 2 (2) |
| *Bradyrhizobium* sp. V  (3 isolates) | H20 | YIC1741 | 2 | Rushan (2) |
|  | H21 | YIC1840 | 1 | Rushan (1) |
| *Bradyrhizobium* sp. VI | H22 | YIC2820 | 1 | Penglai (1) |
| *B. lablabi* | H23 | YIC1859 | 3 | Laohan (3) |
| *Bradyrhizobium* sp. VII | H24 | YIC1466 | 26 | Laoshan (1), Yantai (9), Rongcheng 1 (4), Rongcheng 2 (6), Rushan (2), Haoyang (4) |
| *Bradyrhizobium* sp. VIII | H25 | YIC1771 | 2 | Laoshan (2) |
| *Bradyrhizobium* sp. IX | H26 | YIC1983 | 1 | Weihai (1) |
| *B. elkanii* (433 isolates) | H27 | YIC1805 | 108 | Weihai (21), Rongcheng 1 (4), Rongcheng 2 (13), Rushan (4), Haiyang (4), Jimo (15), Qingdao (11), Penglai (3), Yantai (33) |
|  | H28 | YIC1970 | 53 | Weihai (25), Rongcheng 1 (7), Rongcheng 2 (4), Rushan (10), Jimo (3), Yantai (4) |
|  | H29 | YIC3174 | 2 | Jimo (1), Rizhao (1) |
|  | H30 | YIC1926 | 238 | Weihai (5), Rongcheng 1 (28), Rongcheng 2 (28), Rushan (19), Haiyang (1), Jimo (21), Laoshan (26), Qingdao (14), Rizhao (57), Penglai (6), Yantai (33) |
|  | H31 | YIC2861 | 32 | Weihai (2), Rongcheng 1 (8), Rongcheng 2 (1), Rushan (5), Jimo (4), Laoshan (6), Qingdao (1), Penglai (2), Yantai (3) |
| *B. ferriligni* (226 isolates) | H32 | YIC1786 | 17 | Rongcheng 1 (3), Rongcheng 2 (1), Jimo (2), Laoshan (9), Yantai (2) |
|  | H33 | YIC3667 | 116 | Weihai (2), Rongcheng 1 (6), Rongcheng 2 (26), Rushan (5), Haiyang (24), Laoshan (10), Qingdao (15), Penglai (21), Yantai (7) |
|  | H34 | YIC1881 | 79 | Weihai (2), Rongcheng 1 (15), Rongcheng 2 (7), Rushan (10), Haiyang (1), Jimo (9), Laoshan (12), Qingdao (3), Penglai (6), Yantai (14) |
|  | H35 | YIC3886 | 14 | Rongcheng 1 (1), Rushan (2), Jimo (5), Laoshan (1), Qingdao (1), Penglai (2), Yantai (2) |
| *B. pachyrhizi* (81 isolates) | H36  H37  H38  H39  H40  H41 | YIC1782  YIC1998  YIC1719  YIC2967  YIC1789  YIC1966 | 12  9  38  1  10  11 | Rongcheng 2 (9), Rushan (2), Yantai (1)  Penglai (1), Rongcheng1 (1), Rongcheng2 (3),Rushan (1), Jimo (1),Rizhao (2)  Weihai (2), Rongcheng1 (2), Rongcheng2 (7),  Rushan (6), Haiyang (4), Jimo (2), Laoshan (12), Qingdao (3)  Laoshan (1)  Yantai (3), Rongcheng1 (3), Rushan (1), Jimo (2), Laoshan (1)  Yantai (2), Rongcheng2 (3), Rushan (2), Jimo (1), Laoshan (3) |

**Table S2**. Distribution of rhizobia in different sampling sites and rhizobial haplotype classification

| **Isolates (YIC code)** | **recA haplotype** | **Species affiliation** |
| --- | --- | --- |
| Isolates from Penglai |  |  |
| 1803, 1804, 1816, 1872, 1873, 1876, 1944, 1945 | H18 | *B. ottawaense* |
| 2820 | H22 | *Bradyrhizobium* sp. VI |
| 1805, 1948, 2862 | H27 | *B. elkanii* |
| 1808, 1997, 2344, 2345, 2577, 2841 | H30 |  |
| 1878, 1879 | H31 |  |
| 1810, 1812, 1814, 1815, 1870, 1871, 1877, 1990, 2343, 2414, 2415, 2422, 2529, 2531, 2561, 2793, 2811, 2821, 2842, 2865, 2960 | H33 | *B. ferriligni* |
| 1807, 1813, 1874, 1875, 1880, 2501 | H34 |  |
| 1999, 2789 | H35 |  |
| 2840 | H37 | *B. pachyrhizi* |
| Isolates from Yantai |  |  |
| 1796, 1797, 1892, 1893, 1820, 1823, 2266, 2267, 2350, 2535 | H7 | *Bradyrhizobium* sp. II |
| 2281 | H8 |  |
| 1074 | H10 | *B. daqingense* |
| 1123 | H11 | *B. liaoningense* |
| 1081 | H12 |  |
| 1053, 1059, 1061, 1054, 1055, 1060, 1080, 1134, 1157 | H24 | *Bradyrhizobium* sp. VII |
| 1781, 1821, 1827, 1968, 1969, 1971, 1973, 1974, 1975, 2268, 2279, 2280, 2300, 2363, 2364, 2365, 2370, 2377, 2533, 2536, 2541, 2546, 2558, 2571, 2584, 2626, 2629, 2852, 2859, 2860, 3619, 1082, 1151 | H27 | *B. elkanii* |
| 1970, 2614, 1117, 1120 | H28 |  |
| 1783, 1819, 1882, 1914, 1926, 1930, 1931, 1965, 2272, 2274, 2310, 2319, 2334, 2339, 2346, 2521, 2522, 2523, 2524, 2525, 2528, 2549, 2555, 2838, 1118, 1125, 1136, 1079, 1109, 1119, 1132, 1138, 1156 | H30 |  |
| 2556, 2861, 1154 | H31 |  |
| 1795, 1786 | H32 | *B. ferriligni* |
| 1972, 2545, 2788, 1058, 1135, 1071, 1078 | H33 |  |
| 1787, 1788, 1790, 1791, 1792, 1794, 1809, 2824, 1780, 1881, 2292, 2544, 1052, 1137 | H34 |  |
| 2582, 2863 | H35 |  |
| 1782 | H36 | *B. pachyrhizi* |
| 1784, 1785, 1789 | H40 |  |
| 1966, 1967 | H41 |  |
| Isolates from Weihai |  |  |
| 3617 | H6 | *Bradyrhizobium* sp. I |
| 2342, 2439, 2514, 2785, 2855, 3526, 3928 | H7 | *Bradyrhizobium* sp. II |
| 2465, 2512 | H15 | *Bradyrhizobium* sp. IV |
| 3320 | H18 | *B. ottawaense* |
| 1983 | H26 | *Bradyrhizobium* sp. IX |
| 1700, 1701, 1703, 1704, 1749, 1752, 1754, 1755, 1756, 1757, 1940, 2311, 2325, 2331, 2332, 2340, 2353, 2354, 2392, 2500, 2835 | H27 | *B. elkanii* |
| 1689, 1690, 1691, 1692, 1694, 1695, 1698, 1699, 1751, 1759, 1760, 2366, 2436, 2464, 2570, 2589, 2813, 2814, 2816, 2839, 2959, 3312, 3321, 3659, 3925 | H28 |  |
| 1696, 1750, 2352, 2445, 2466 | H30 |  |
| 1753, 1758 | H31 |  |
| 1688, 2823 | H33 | *B. ferriligni* |
| 2367, 2369 | H34 |  |
| 1693, 1697 | H38 | *B. pachyrhizi* |
| Isolates from Rongcheng 1 |  |  |
| 2579, 3578, 3946 | H7 | *Bradyrhizobium* sp. II |
| 2845 | H9 | *Bradyrhizobium* sp. III |
| 2607 | H12 | *B. liaoningense* |
| 2931 | H17 | *B. arachidis* |
| 3587, 1982, 2409, 2413 | H24 | *Bradyrhizobium* sp. VII |
| 1730, 2324, 1908, 2400 | H27 | *B. elkanii* |
| 2263, 2484, 2818, 2828, 2864, 2866, 3603 | H28 |  |
| 1731, 2273, 2318, 2388, 2402, 2617, 1923, 1981, 1985, 2261, 2270, 2271, 2276, 2378, 2405, 2408, 2410, 2481, 2483, 2486, 2487, 2491, 2494, 2853, 2856, 2889, 2951, 2964 | H30 |  |
| 2312, 2313, 2613, 1906, 2488, 2504, 2518, 2822 | H31 |  |
| 2306, 2519, 2825 | H32 | *B. ferriligni* |
| 1733, 2316, 2317, 2349, 2262, 2440 | H33 |  |
| 1732, 1910, 1911, 1912, 1915, 1922, 1929, 1984, 2335, 2336, 2616, 2832, 2892, 1907, 2509 | H34 |  |
| 2393 | H35 |  |
| 2963 | H37 | *B. pachyrhizi* |
| 1909, 2817 | H38 |  |
| 2401, 2482, 2808 | H40 |  |
| Isolates from Rongcheng 2 |  |  |
| 2068 | H7 | *Bradyrhizobium* sp. II |
| 2166 | H9 | *Bradyrhizobium* sp. III |
| 2168, 2180, 2576 | H15 | *Bradyrhizobium* sp. IV |
| 2164, 2210, 2550, 2567 | H18 | *B. ottawaense* |
| 2235, 1835 | H19 |  |
| 2044, 2403, 2538, 2568, 2620, 2624 | H24 | *Bradyrhizobium* sp. VII |
| 2067, 2189, 2193, 2211, 2212, 2224, 2226, 2227, 2228, 2229, 2239, 2240, 2251 | H27 | *B. elkanii* |
| 2223, 1799, 2283, 2621 | H28 |  |
| 2066, 64285, 64316, 2081, 2083, 69399, 69430, 2091, 2092, 2169, 2170, 2187, 2188, 2230, 2231, 2256, 2258, 1798, 1833, 1834, 2285, 2347, 2411, 2511, 2517, 2787, 2831, 2945 | H30 |  |
| 2099 | H31 |  |
| 2165 | H32 | *B. ferriligni* |
| 2138, 2139, 2141, 2142, 2181, 2182, 2190, 2192, 2195, 2201, 2204, 2205, 2209, 2225, 2232, 2233, 2234, 2237, 2241, 2250, 2257, 1836, 1837, 2275, 2348, 3667 | H33 |  |
| 2191, 2206, 1800, 1832, 2291, 2562, 2802 | H34 |  |
| 2074-1, 63586, 2202, 2203, 2207, 2208, 2213, 2238, 2249 | H36 | *B. pachyrhizi* |
| 2252, 2253, 1998 | H37 |  |
| 2167, 2186, 2236, 2284, 2553, 2575, 2956 | H38 |  |
| 1811, 1830, 2559 | H41 |  |
| Isolates from Rushan |  |  |
| 1839, 2471, 2472 | H16 | *B. arachidis* |
| 1740, 1741 | H20 | *Bradyrhizobium* sp. V |
| 1840 | H21 |  |
| 1838, 1847 | H24 | *Bradyrhizobium* sp. VII |
| 1738, 1848, 2278, 2470 | H27 | *B. elkanii* |
| 1745, 1746, 1747, 1900, 1901, 1902, 2441, 2548, 2583, 2623 | H28 |  |
| 1736, 1743, 1748, 1844, 1896, 2259, 2265, 2391, 2397, 2398, 2399, 2406, 2407, 2469, 2551, 2569, 2585, 2882, 3947 | H30 |  |
| 1735, 1737, 1843, 1895, 2473 | H31 |  |
| 1845, 1897, 2327, 2438, 2883 | H33 | *B. ferriligni* |
| 1828, 1829, 1842, 1846, 1894, 1899, 1925, 2351, 2396, 2586 | H34 |  |
| 2404, 3669 | H35 |  |
| 1744, 2394 | H36 | *B. pachyrhizi* |
| 1898 | H37 |  |
| 1739, 1841, 2277, 2479, 2796, 3576 | H38 |  |
| 1742 | H40 |  |
| 2395, 2792 | H41 |  |
| Isolates from Haiyang |  |  |
| 1441, 1443, 1444, 1490, 1537 | H1 | *B. yuanmingense* |
| 1439 | H2 |  |
| 1482, 1486, 1488 | H3 |  |
| 1538 | H4 |  |
| 1480 | H5 |  |
| 1514 | H7 | *Bradyrhizobium* sp. II |
| 1438 | H11 | *B. liaoningense* |
| 1445 | H12 |  |
| 1477 | H13 |  |
| 1442, 1481, 1484, 1505, 1509, 1518, 1535 | H14 |  |
| 1465, 1466, 1470, 1473 | H24 | *Bradyrhizobium* sp. VII |
| 1479, 1483, 1536, 1539 | H27 | *B. elkanii* |
| 1507 | H30 |  |
| 1471, 1472, 1475, 1476, 1478, 1487, 1489, 1491, 1502, 1504, 1506, 1508, 1510, 1511, 1512, 1513, 1515, 1516, 1517, 1519, 1520, 1521, 1534, 1545 | H33 | *B. ferriligni* |
| 1503 | H34 |  |
| 1485, 1500, 1501, 1540 | H38 | *B. pachyrhizi* |
| Isolates from Jimo |  |  |
| 1904, 1918, 1919, 1946, 2515, 2526, 2539, 2580 | H15 | *Bradyrhizobium* sp. IV |
| 1947, 1949, 1950, 1951, 2289, 2437, 2442, 2443, 2458, 2497, 2520, 2578, 2610, 2625, 2628 | H27 | *B. elkanii* |
| 2587, 2611, 2827 | H28 |  |
| 2506 | H29 |  |
| 1987, 1988, 2450, 2451, 2452, 2454, 2457, 2460, 2463, 2496, 2543, 2547, 2552, 2630, 2631, 2884, 2927, 2929, 2944, 2950, 2977 | H30 |  |
| 1986, 2480, 2887, 2888 | H31 |  |
| 2456, 2554 | H32 | *B. ferriligni* |
| 1905, 1917, 1920, 1921, 2453, 2462, 2493, 2588, 2930 | H34 |  |
| 2459, 2461, 2495, 2786, 3886 | H35 |  |
| 2455 | H37 | *B. pachyrhizi* |
| 2846, 2962 | H38 |  |
| 1933, 2527 | H40 |  |
| 2948 | H41 |  |
| Isolates from Laoshan |  |  |
| 1718 | H5 | *B. yuanmingense* |
| 1851, 1856, 1913, 1941, 2290, 2320 | H7 | *Bradyrhizobium* sp. II |
| 2326 | H10 | *B. daqingense* |
| 2314 | H15 | *Bradyrhizobium* sp. IV |
| 1705 | H18 | *B. ottawaense* |
| 1773, 1824, 1859 | H23 | *B. lablabi* |
| 1854 | H24 | *Bradyrhizobium* sp. VII |
| 1771, 2502 | H25 | *Bradyrhizobium* sp. VIII |
| 1707, 1710, 1712, 1715, 1716, 1717, 1724, 1725, 1727, 1857, 1858, 1916, 1924, 1932, 2260, 2269, 2282, 2293, 2315, 2322, 2337, 2376, 2485, 2498, 2507, 2815 | H30 | *B. elkanii* |
| 1706, 1723, 1726, 1927, 1928, 1903 | H31 |  |
| 1852, 1761, 1762, 1763, 1825, 1863, 1864, 1869, 2321 | H32 |  |
| 1708, 1713, 1714, 1722, 1729, 1853, 2287, 2323, 2341, 2797 | H33 |  |
| 1850, 1855, 1942, 1765, 1770, 1774, 1775, 1779, 1861, 1868, 2328, 2355 | H34 | *B. ferriligni* |
| 1777 | H35 |  |
| 1711, 1719, 1764, 1768, 1776, 1778, 1822, 1860, 1866, 2826, 2849, 2885 | H38 | *B. pachyrhizi* |
| 2967 | H39 |  |
| 1709 | H40 |  |
| 1728, 1943, 1867 | H41 |  |
| Isolates from Qingdao |  |  |
| 3100 | H1 | *B. yuanmingense* |
| 3200, 3201 | H4 |  |
| 3118, 3283 | H7 | *Bradyrhizobium* sp. II |
| 3117, 3127, 3184, 3191, 3194, 3199, 3208, 3210, 3211, 3252, 3293 | H27 | *B. elkanii* |
| 3121, 3122, 3123, 3188, 3192, 3193, 3195, 3197, 3198, 3202, 3204, 3206, 3251, 3285 | H30 |  |
| 3288 | H31 |  |
| 3101, 3102, 3103, 3105, 3106, 3185, 3186, 3189, 3190, 3205, 3212, 3282, 3289, 3290, 3292 | H33 | *B. ferriligni* |
| 3203, 3286, 3305 | H34 |  |
| 3287 | H35 |  |
| 3099, 3209, 3253 | H38 | *B. pachyrhizi* |
| Isolates from Rizhao |  |  |
| 3174 | H29 | *B. elkanii* |
| 3137, 3138, 3139, 3140, 3141, 3142, 3143, 3144, 3145, 3146, 3148, 3149, 3150, 3151, 3152, 3153, 3154, 3155, 3156, 3157, 3158, 3159, 3160, 3161, 3162, 3163, 3164, 3165, 3166, 3167, 3168, 3169, 3170, 3171, 3172, 3173, 3175, 3176, 3177, 3178, 3179, 3232, 3233, 3234, 3235, 3236, 3237, 3238, 3239, 3240, 3241, 3242, 3243, 3275, 3276, 3303, 3304 | H30 |  |
| 3147, 3302 | H37 | *B. pachyrhizi* |

**Table S3**. List of the accession numbers obtained in this study.

| Strains | *dnaK* | *glnII* | *gyrB* | *recA* | *ropB* | *nifH* | *nodC* |
| --- | --- | --- | --- | --- | --- | --- | --- |
| YIC1438 | MW511634 | MW511552 | MW511675 | MW528316 | MW511593 | MW511718 | MW511762 |
| YIC1439 | MW511635 | MW511553 | MW511676 | MW528317 | MW511594 | MW511719 | MW511763 |
| YIC1445 | MW511672 | MW511592 | MW511715 | MW528356 | MW511633 | MW511720 | MW511764 |
| YIC1466 | MW511674 | MW511591 | MW511714 | MW528355 | MW511632 | MW511738 | MW511761 |
| YIC1477 | MW511636 | MW511554 | MW511677 | MW528318 | MW511595 | MW511721 | MW511765 |
| YIC1480 | MW511637 | MW511555 | MW511678 | MW528319 | MW511596 | MW511722 | MW511766 |
| YIC1484 | MW511638 | MW511556 | MW511679 | MW528320 | MW511597 | MW511723 | MW511767 |
| YIC1488 | MW511639 | MW511557 | MW511680 | MW528321 | MW511598 | MW511724 | MW511768 |
| YIC1537 | MW511640 | MW511558 | MW511681 | MW528322 | MW511599 | MW511725 | MW511769 |
| YIC1538 | MW511641 | MW511559 | MW511682 | MW528323 | MW511600 | MW511726 | MW511770 |
| YIC1719 | MW511642 | MW511560 | MW511683 | MW528324 | MW511601 | MW511727 | MW511771 |
| YIC1741 | MW511643 | MW511561 | MW511684 | MW528325 | MW511602 | MW511728 | MW511772 |
| YIC1771 | MW511644 | MW511562 | MW511685 | MW528326 | MW511603 | MW511729 | MW511773 |
| YIC1782 | MW511645 | MW511563 | MW511686 | MW528327 | MW511604 | MW511730 | MW511774 |
| YIC1786 | MW511646 | MW511564 | MW511687 | MW528328 | MW511605 | MW511731 | MW511775 |
| YIC1789 | MW511647 | MW511565 | MW511688 | MW528329 | MW511606 | MW511732 | MW511776 |
| YIC1803 | MW511648 | MW511566 | MW511689 | MW528330 | MW511607 | MW511733 | MW511777 |
| YIC1805 | MW511649 | MW511567 | MW511690 | MW528331 | MW511608 | MW511734 | MW511778 |
| YIC1835 | MW511650 | MW511568 | MW511691 | MW528332 | MW511609 | MW511735 | MW511779 |
| YIC1839 | MW511651 | MW511569 | MW511692 | MW528333 | MW511610 | MW511736 | MW511780 |
| YIC1840 | MW511652 | MW511570 | MW511693 | MW528334 | MW511611 | MW511737 | MW511781 |
| YIC1859 | MW511653 | MW511571 | MW511694 | MW528335 | MW511612 | MW511716 | MW511758 |
| YIC1881 | MW511654 | MW511572 | MW511695 | MW528336 | MW511613 | MW511739 | MW511782 |
| YIC1913 | MW511655 | MW511573 | MW511696 | MW528337 | MW511614 | MW511717 | MW511784 |
| YIC1919 | MW511656 | MW511574 | MW511697 | MW528338 | MW511615 | MW511740 | MW511785 |
| YIC1926 | MW511657 | MW511575 | MW511698 | MW528339 | MW511616 | MW511741 | MW511786 |
| YIC1966 | MW511658 | MW511576 | MW511699 | MW528340 | MW511617 | MW511742 | MW511787 |
| YIC1970 | MW511659 | MW511577 | MW511700 | MW528341 | MW511618 | MW511743 | MW511760 |
| YIC1983 | MW511660 | MW511578 | MW511701 | MW528342 | MW511619 | MW511744 | MW511788 |
| YIC1998 | MW511661 | MW511579 | MW511702 | MW528343 | MW511620 | MW511745 | MW511789 |
| YIC2281 | MW511662 | MW511580 | MW511703 | MW528344 | MW511621 | MW511746 | -- |
| YIC2326 | MW511663 | MW511581 | MW511704 | MW528345 | MW511622 | MW511747 | -- |
| YIC2820 | MW511664 | MW511582 | MW511705 | MW528346 | MW511623 | MW511748 | MW511757 |
| YIC2845 | MW511665 | MW511583 | MW511706 | MW528347 | MW511624 | MW511749 | MW511790 |
| YIC2861 | MW511666 | MW511584 | MW511707 | MW528348 | MW511625 | MW511750 | MW511759 |
| YIC2931 | MW511667 | MW511585 | MW511708 | MW528349 | MW511626 | MW511751 | MW511791 |
| YIC2967 | MW511668 | MW511586 | MW511709 | MW528350 | MW511627 | MW511752 | MW511792 |
| YIC3174 | MW511669 | MW511587 | MW511710 | MW528351 | MW511628 | MW511753 | MW511793 |
| YIC3617 | MW511670 | MW511588 | MW511711 | MW528352 | MW511629 | MW511754 | MW511794 |
| YIC3667 | MW511673 | MW511590 | MW511713 | MW528354 | MW511631 | MW511755 | MW511783 |
| YIC3886 | MW511671 | MW511589 | MW511712 | MW528353 | MW511630 | MW511756 | MW511795 |

**Table S4.** Taxonomy and *recA*, *dnaK*, *gyrB*, *rpoB*, *glnII* and MLSA similarities of intra-genospecies isolated in this study, and MLSA similarities between each haplotype with the related published species.

| Representative strains | Similarities between haplotypes of intra-genospecies | | | | | | MLSA Similarities between haplotypes and related species | Definition of genospecies |
| --- | --- | --- | --- | --- | --- | --- | --- | --- |
|  | recA | dnaK | gyrB | rpoB | glnII | MLSA |  |  |
| YIC1439, YIC1480, YIC1488, YIC1537, YIC1538 | 96.1-98.6% | 98.4-99.8% | 96.8-99.5% | 99.0-100% | 98.3-100% | 98.1-98.9% | 98.2-99.8%, *B. yuanmingense* CCBAU 10071^T^ | *B. yuanmingense* |
| YIC3617 |  |  |  |  |  |  | 96.8%, *B. yuanmingense* CCBAU 10071^T^ | *Bradyrhizobium* sp. I |
| YIC1913, YIC2281 | 97.6% | 99.3% | 100.0% | 99.4% | 97.6% | 98.8% | 96.3%, *B. yuanmingense* CCBAU 10071^T^ | *Bradyrhizobium* sp. II |
| YIC2845 |  |  |  |  |  |  | 95.8%, *B. yuanmingense* CCBAU 10071^T^ | *Bradyrhizobium* sp. III |
| YIC2326 |  |  |  |  |  |  | 99.4%, *B. daqingense* CCBAU 15774^T^ | *B. daqingense* |
| YIC1438, YIC1445, YIC1477, YIC1484 | 96.8-99.5% | 99.1-99.8% | 97.9-99.7% | 99.0-100% | 94.5-99.3% | 98.2-99.7% | 97.9-99.0%, *B. liaoningense* USDA 3622^T^ | *B. liaoningense* |
| YIC1919 |  |  |  |  |  |  | 95.6%, *B. liaoningense* USDA 3622^T^ | *Bradyrhizobium* sp. IV |
| YIC1839, YIC2931 | 98.6% | 100.0% | 100.0% | 99.1% | 88.4% | 97.2% | 95.6-97.8%, *B. arachidis* CCBAU 051107^T^ | *B. arachidis* |
| YIC1803, YIC1835 | 99.8% | 100.0% | 100.0% | 100.0% | 97.8% | 99.5% | 99.1-99.7%, *B. ottawaense* OO85^T^ | *B. ottawaense* |
| YIC1741, YIC1840 | 99.1% | 98.4% | 92.4% | 99.1% | 99.0% | 97.6% | 96.8-96.9%, *B. japonicu*m LMG 6138^T^ | *Bradyrhizobium* sp. V |
| YIC2820 |  |  |  |  |  |  | 96.3%, *B. lablabi* CCBAU 23086^T^ | *Bradyrhizobium* sp. VI |
| YIC1859 |  |  |  |  |  |  | 97.5%, *B. lablabi* CCBAU 23086^T^ | *B. lablabi* |
| YIC1466 |  |  |  |  |  |  | 96.2%, *B. elkanii* USDA 76^T^ | *Bradyrhizobium* sp. VII |
| YIC1771 |  |  |  |  |  |  | 95.8%, *B. embrapense* SEMIA 6208^T^ | *Bradyrhizobium* sp. VIII |
| YIC1983 |  |  |  |  |  |  | 96.2%, *B. embrapense* SEMIA 6208^T^ | *Bradyrhizobium* sp. IX |
| YIC1805, YIC1970, YIC3174, YIC1926, YIC2861 | 95.5-99.5% | 98.9-100% | 99.0-100% | 98.9-99.6% | 99.5-100% | 98.7-99.7% | 98.8-99.4%, *B. elkanii* USDA 76^T^ | *B. elkanii* |
| YIC1786, YIC3667, YIC1881, YIC3886 | 98.4-99.8% | 99.3-100% | 96.3-99.7% | 99.0-100% | 99.1-100% | 98.6-99.9% | 98.0-98.3%, *B. ferriligni* CCBAU 51502^T^ | *B. ferriligni* |
| YIC1782, YIC1998, YIC1719, YIC2967 YIC1789, YIC1966 | 96.4-99.5% | 98.4-100% | 98.2-100% | 99.3-100% | 98.8-100% | 98.4-99.5% | 98.1-98.3%, *B. pachyrhizi* LMG 24246^T^ | *B. pachyrhizi* |

**Table S5.** Different haplotype numbers were observed through analyses using different housekeeping gene sequences of 41 representative strains.

| Gene | Haplotype number |
| --- | --- |
| *recA* | 41 |
| *dnaK* | 34 |
| *glnII* | 33 |
| *gyrB* | 35 |
| *rpoB* | 35 |
